# Differential responses of MET activations to MET kinase inhibitor and neutralizing antibody

**DOI:** 10.1186/s12967-018-1628-y

**Published:** 2018-09-12

**Authors:** Jianqun Kou, Phillip R. Musich, Ben Staal, Liang Kang, Yuan Qin, Zhi Q. Yao, Boheng Zhang, Weizhong Wu, Angela Tam, Alan Huang, Huai-Xiang Hao, George F. Vande Woude, Qian Xie

**Affiliations:** 1 Department of Biomedical Sciences, Quillen College of Medicine, East Tennessee State University, Johnson City, TN 37614 USA; 2 Center of Excellence for Inflammation, Infectious Disease and Immunity, Quillen College of Medicine, East Tennessee State University, Johnson City, TN 37614 USA; 3 Center of Cell and Cancer Biology, Van Andel Research Institute, Grand Rapids, MI 49503 USA; 4 Department of Internal Medicine, Quillen College of Medicine, East Tennessee State University, Johnson City, TN 37614 USA; 5 Liver Cancer Institute, Zhongshan Hospital, Fudan University, Shanghai, 200032 China; 6 Novartis Institutes for BioMedical Research, Cambridge, MA 02139 USA

**Keywords:** Hepatocyte growth factor, Met, Tyrosine kinase inhibitor, Neutralizing antibody, Targeted therapy, Combination therapy

## Abstract

**Background:**

Aberrant MET tyrosine kinase signaling is known to cause cancer initiation and progression. While MET inhibitors are in clinical trials against several cancer types, the clinical efficacies are controversial and the molecular mechanisms toward sensitivity remain elusive.

**Methods:**

With the goal to investigate the molecular basis of MET amplification (MET^*amp*^) and hepatocyte growth factor (HGF) autocrine-driven tumors in response to MET tyrosine kinase inhibitors (TKI) and neutralizing antibodies, we compared cancer cells harboring MET^*amp*^ (MKN45 and MHCCH97H) or HGF-autocrine (JHH5 and U87) for their sensitivity and downstream biological responses to a MET-TKI (INC280) and an anti-MET monoclonal antibody (MetMab) in vitro, and for tumor inhibition in vivo.

**Results:**

We find that cancer cells driven by MET^amp^ are more sensitive to INC280 than are those driven by HGF-autocrine activation. In MET^amp^ cells, INC280 induced a DNA damage response with activation of repair through the p53BP1/ATM signaling pathway. Although MetMab failed to inhibit MET^amp^ cell proliferation and tumor growth, both INC280 and MetMab reduced HGF-autocrine tumor growth. In addition, we also show that HGF stimulation promoted human HUVEC cell tube formation via the Src pathway, which was inhibited by either INC280 or MetMab. These observations suggest that in HGF-autocrine tumors, the endothelial cells are the secondary targets MET inhibitors.

**Conclusions:**

Our results demonstrate that MET^*amp*^ and HGF-autocrine activation favor different molecular mechanisms. While combining MET TKIs and ATM inhibitors may enhance the efficacy for treating tumors harboring MET^amp^, a combined inhibition of MET and angiogenesis pathways may improve the therapeutic efficacy against HGF-autocrine tumors.

**Electronic supplementary material:**

The online version of this article (10.1186/s12967-018-1628-y) contains supplementary material, which is available to authorized users.

## Background

Aberrant activation of MET signaling is significantly correlated with cancer malignancy and poor clinical outcomes [1–3]. While overexpression of hepatocyte growth factor (HGF) by stromal or tumor cells may result in ligand-dependent MET activation in a paracrine or autocrine manner [4, 5], focal amplification of the MET receptor gene (MET^amp^) [6, 7], its point mutation (MET^mut^) [8, 9], or alternative splicing of its mRNA [10, 11] also may cause a ligand-independent MET activation. In addition, MET signaling can be cross-activated by other signaling pathways such as EGFR [12], VEGF [13] and WNT [14]. Upon stimulation, MET induces a cellular program known as invasive growth, which promotes proliferation, motility, invasion, and morphogenesis [1]. As MET activation is shown to drive cancer initiation, progression, and resistance to chemotherapeutics, targeting MET signaling has become a promising strategy for cancer treatment [2].

Recent MET inhibitors under clinical development are mainly tyrosine kinase inhibitors (TKIs) or neutralizing antibodies. MET TKIs are classified further into selective and non-selective inhibitors, or ATP and non-ATP competitors, while monoclonal antibodies are developed to target either HGF or MET. Extensive studies have shown that tumors with MET^mut^ [10, 11], MET^amp^ [4, 6], HGF-autocrine [4, 12, 15] or paracrine activation [5] are sensitive to MET inhibitors; however, clinical trial results with MET inhibitors are not consistent. In renal cancer, phase II trial of savolitinib, a highly selective MET TKI, has shown promising activity and tolerability in a subset of patients with MET-driven advanced papillary renal cell carcinoma (PRCC). Thus, MET is becoming a potential target across all papillary renal cell carcinomas [16, 17]. However, the efficacy of MET inhibitors remains undocumented in other types of cancer. In non-small cell lung cancer (NSCLC), MET-targeted therapeutics only improves progression-free survival (PFS) but provides no benefit for overall survival (OS) [18]. In glioblastoma patients, while cabozantinib (XL184), a non-selective, ATP competitor targeting MET, VEGFR2, and AXL demonstrated encouraging clinical efficacy in a phase II trial in glioblastoma (GBM) [19–21], rilotumumab, an HGF antibody, showed a lack of efficacy, possibly due to the selection of patients who were heavily pretreated by other therapeutics [22]. With advanced hepatocellular carcinoma (HCC), while tivantinib (ARQ 197) and cabozantinib show efficacy in phase II/III trials [23, 24], both are multi-target Met inhibitors with unknown long-term efficacy and toxicity. Other inhibitors in clinical trials also include INC280, a selective TKI, and the antibody emibetuzumab [25, 26]. Given that many MET inhibitors are entering clinical trials there is a critical need to develop a patient stratification strategy for precisely selecting patients who may benefit from MET-targeted therapies and to explore the molecular basis of response to MET-targeting therapeutics to improve efficacy [2, 27].

We previously reported that tumors harboring MET^*amp*^ or HGF-autocrine activation are vulnerable to MET inhibitors in HCC [4] and GBM [12]. In this study, we further elucidated the distinct mechanisms defining these two types of MET oncogenic activation, and their differential therapeutic responses to the specific MET TKI, INC280 and the neutralizing antibody MetMab. We show that MET^*amp*^ is prone to INC280 inhibition through a DNA damage response (DDR) and repair mechanism, likely due to a double-strand break (DSB). In HGF-autocrine tumors, tumor-derived HGF may promote angiogenesis via promoting vasculature formation by endothelial cells. As such, the endothelial cells are the second “hit” by either INC280 or MetMab (see summary Fig. 6). Our results suggest that different MET oncogenic activations may lead to differential therapeutic responses, which warrants further evaluation in future clinical trials of MET inhibitors and in the design of combination strategies.

## Methods

### Cell lines and drugs

Human cancer cells MKN45 (gastric) and U87 (glioma) were obtained from American Tissue Type Collection (ATCC); JHH5 (hepatocellular carcinoma) was obtained from the Japanese Collection of Research Bioresources (JCRB). MHCC97H was provided by Fudan University Liver Cancer Institute [4]. Human endothelial cells HUVEC were purchased from Lonza. Briefly, the MKN45 cell line was grown in RPMI-1640 supplemented with 10% FBS. MHCC97H, JHH5 and U87 cells were grown in DMEM with 10% FBS. HUVEC cells were maintained in EGM-2 medium and subjected to EBM-2 basal medium prior to the tube formation assay (Lonza). INC280 is a MET TKI provided by Novartis. MetMab (onartuzumab) is produced in CHO cells at Novartis according to published patent US 2011/0262436 for research use only. KU60019 is a specific ATM inhibitor purchased from Abcam. INC280 and KU60019 compounds were dissolved in DMSO at 0.01 M and aliquots were stored at − 80 °C until use. MetMab stock was in PBS and kept at 4 °C until use. To treat cells in vitro, stock solutions were serially diluted using culture medium as indicated above.

### CellTiter-Glo assay

Cells were seeded into a 96-well plate at 5 × 10^3^ cells/well and grown overnight at 37 °C followed by treatment with INC280 or MetMab at the indicated concentrations. Triplicate wells were used for each concentration. After an additional 72 h, CellTiter-Glo reagent was added into each well and incubation continued for 10 min at room temperature following the manufacturer’s instructions (Promega). Luminescence signal intensity was measured by a microplate reader (BioTek).

### Cell cycle analysis

Cells were seeded at 5 × 10^4^ cells/well in 6-well plates and grown until 60–80% confluency. After serum starvation overnight, the medium was changed to DMEM with 10% FBS and the cells were treated by INC280 at different concentrations for 24 h, followed by cell cycle analysis. To examine the sustained cell cycle arrest effect in MKN45 cells, the cells were rinsed and allowed to recover in complete drug-free medium for an additional 24 or 48 h after INC280 treatment. To analyze cell cycle transit, cells were trypsinized, washed by PBS, and fixed in cold 70% ethanol for 18 h before rinsing in PBS and staining with propidium iodide (50 µg/ml) for 10 min. Cell cycle distribution was determined in a flow cytometer (FACSCalibur, BD). The percentage of cells in each cell cycle phase was analyzed using FlowJo software (FlowJo LLC).

### Immunofluorescent staining

Cells were seeded at 5000 cells/well in 6-well chamber slides (Lab-Tek) and grown to 60–80% confluency. After INC280 treatment for 18 h, cells were washed with PBS, fixed with 4% paraformaldehyde, permeabilized in 0.5% Triton X-100 for 20 min, and blocked in 5% BSA for 1 h at room temperature. To perform immunofluorescence (IF) staining, cells were incubated with mouse anti-γH2AX antibody (1:125 in 5% BSA, BioLegend) and rabbit anti-53BP1 antibody [1:100 in 5% BSA, Cell Signaling Technology (CST)] overnight at 4 °C. After washing 3 times in PBS cells were incubated with goat anti-mouse/Alexa488 and anti-rabbit/Alexa568 antibody (1:500 in PBST, Invitrogen) for 2 h. The nuclei were stained with DAPI (1 µg/ml, Thermo). Images were captured using an EVOS fluorescence microscope (Thermal Fisher).

### Signaling pathway analysis and western blot

Cells seeded in 10-cm dishes were grown until 80% confluent. To determine the MET downstream signaling pathway, cells were serum starved overnight and treated with INC280 or MetMab with or without HGF (100 ng/ml) for 20 min at 37 °C. To determine which cell cycle checkpoints and DNA damage repair pathways were activated, cells were treated by INC280 or MetMab for 24 h at 37 °C without serum starvation. The cells were washed twice with ice-cold PBS, and whole-cell lysates were prepared using RIPA buffer (Fisher). The protein concentrations were determined by the DC protein assay (Bio-Rad). Equal amounts of total protein (30 µg) from cell lysates were loaded on a 4–20% SDS-PAGE gel (Invitrogen), transferred to a polyvinylidene difluoride (PVDF) membrane (Invitrogen), and antibody-protein complexes detected using an ECL Western Blotting Detection System (Thermo). We used antibodies against human Met (clone 25H2), phospho-Met (Y1234/1235), AKT, phospho-AKT (S473), p42/44 MAPK, phospho-p42/44 MAPK (T202/Y204), Gab1, phospho-Gab1 (Y627), CDK4 (D9G3E), CyclinD3 (DCS22), p27 (D69C12), phospho-ATM (Ser1981), phospho-Chk2 (Thr68), phospho-ATR (Ser468), phospho-Chk1 (Ser345), Src, phosphor-Src (Tyr416), p85 (all from CST); γH2AX (BioLegend); β-actin (clone AC-15, Abcam); and HGF (Clone 7-2, Novus). Secondary antibodies used were goat anti-rabbit IgG-HRP and goat anti-mouse IgG-HRP (Santa Cruz Biotechnology).

### Therapeutic efficacy of INC280 and MetMab against tumor growth in *SCID* and *SCIDhgf* mice

Animal studies were approved by the IACUC and conducted at Van Andel Research Institute. Subcutaneous tumor initiation was performed as previously described [4]. Briefly, 5 × 10^5^ cells in 100 μl PBS were injected into the flank of the mice. Dosing with INC280 at 30 mg/kg was delivered once daily by oral gavage for 3 weeks. The vehicle was 0.5% MC 400 with 0.05% Tween 80. MetMab dosing (5, 30 mg/kg) was delivered using a one-time intraperitoneal injection with PBS as vehicle. Tumor growth was measured by caliper twice a week. To determine the effectiveness of treatment, the average tumor size of each group from the last measurement was analyzed with the Student’s *t* test (p < 0.05).

### Tube formation assay

Human umbilical vein endothelial cells (HUVECs) were first cultured in EGM-2 medium and then serum starved in EBM-2 basal medium for 24 h to eliminate the effects of growth factors (Lonza). To perform the tube formation assay, HUVEC cells were trypsinized, re-suspended in EBM-2 basal medium and seeded into a Matrigel-coated 24-well plate at a density of 6000 cells/well [28]. Immediately after seeding the HUVEC cells, INC280 or MetMab was added into each well for 30 min, followed by HGF (100 ng/ml) addition. Each treatment has three replicates. After incubating for another 16 h, phase-contrast images of tube formation were captured on the EVOS microscope. Five images were randomly taken from each well. The number of tube formation was quantified using Image J software and is calculated as mean ± SD of the 15 images.

## Results

### Differential response to INC280 and MetMab in MET^amp^ cells

**Fig. 1:**
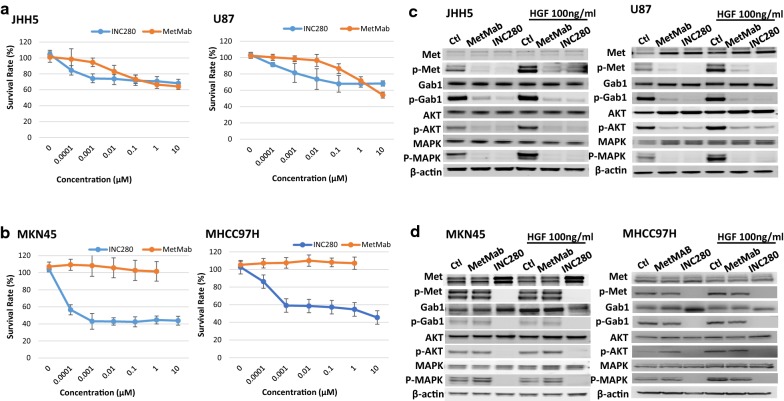
The inhibitory effect of INC280 and MetMab in cancer cell proliferation and the MET signaling pathway. **a**, **b** HGF-autocrine cancer cells JHH5 and U87 (**a**) and MET-amplified cancer cells MKN45 and MHCC97H (**b**) were treated with either INC280 or MetMab for 72 h at various concentrations as indicated. Cell survival was measured using a Cell-titer Glo assay. Survival rate = Luminescence signal intensity of treated samples at indicated concentration/untreated samples. Triplicates were used for each concentration. Vertical bar refers to standard deviation. **c**, **d** Western blots of MET and downstream signaling pathway proteins and the inhibition by INC280 or MetMab in the presence or absence of HGF (100 ng/ml) stimulation in either HGF-autocrine cancer cells (**c**) or MET-amplified cancer cells (**d**). Note that MetMab only inhibited the MET signaling pathway in HGF-autocrine JHH5 and U87 cells regardless of HGF stimulation

Previous studies have shown that MKN45 and MHCC97H cells propagate chromosomal focal amplification in the Met region as determined by FISH analysis [4, 6], and that U87 and JHH5 cells overexpress endogenous HGF and MET accompanied by p-MET, forming an autocrine loop activation [4, 15]. Functionally, both MET^*amp*^ and HGF-autocrine loop drive MET oncogenic activation and these cells are sensitive to MET TKIs such as PHA665752, SGX523, V-4084, and INC280 [4, 6, 12, 15, 29]. To compare the efficacy of a specific MET TKI and neutralizing antibody in inhibiting cancer cells with these two types of MET activation, MKN45, MHCC97H, U87 and JHH5 cells were treated by INC280 or MetMab at various concentrations for 72 h and tested for cell viability using the Cell-titer Glo assay (Fig. 1a, b). We show that both INC280 and MetMab inhibit JHH5 and U87 dose dependently at comparable concentrations (Fig. 1a).  When examining downstream signaling, both JHH5 and U87 cells had endogenous MET activation as indicated by p-MET expression. HGF stimulation further activates p-MET and downstream signaling proteins including p-Gab1, p-AKT, and p-ERK. Phosphorylation of each of these proteins was significantly inhibited by either INC280 or MetMab (Fig. 1c).

In contrast, MKN45 and MHCC97H cells showed significant differences in response to INC280 or MetMab. While INC280 inhibited MKN45 and MHCC97H proliferation more efficiently than in JHH5 and U87, MetMab showed no efficacy in either of these cell lines (Fig. 1b). Western blot analysis of proteins activated in the MET signaling pathway in response to HGF stimulation and MET inhibition by INC280 or MetMab confirm these proliferation assay results (Fig. 1d). We show that both MKN45 and MHCC97H cells expressed a high level of endogenous p-MET and downstream signaling molecules p-Gab1, p-AKT, and p-ERK and that additional HGF stimulation did not further enhance the phosphorylation of MET significantly. INC280, but not MetMab, inhibited phosphorylation of these proteins.

That MetMab inhibits proliferation in JHH5 and U87 cells, but not in MKN45 and MHCC97H cells suggest a differential MET-targeting mechanism between TKIs and ligand-blocking neutralizing antibodies. Because the antibodies block the extracellular HGF/MET binding domain, they only inhibit HGF-dependent MET activation and tumor growth.  By contrast, small molecule TKIs like INC280 compete intracellularly for the ATP binding site in the MET tyrosine kinase domain, thus inhibit the constitutive MET-activation regardless of HGF stimulation.

### MET^amp^ predisposes cells to DNA double-strand breaks with INC280 treatment

**Fig. 2:**
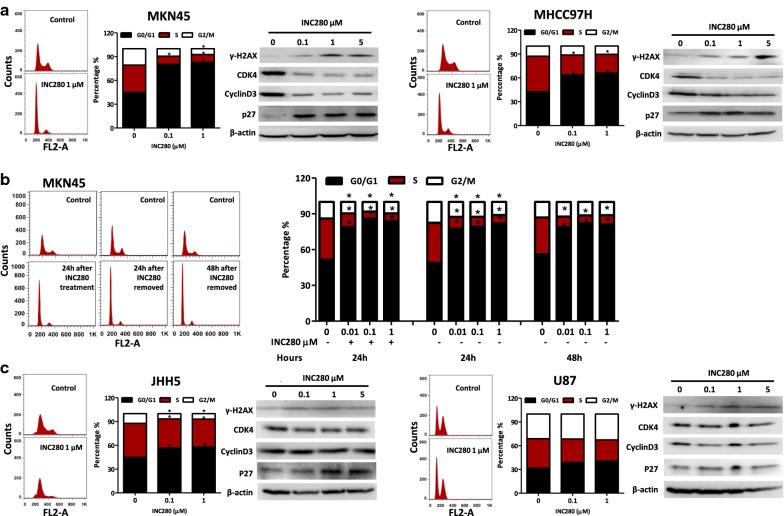
INC280 induces potent cell cycle arrest at G1 phase in MKN45 and MHCC cells. **a** MKN45 and MHCC97H cells were treated with INC280 (0, 0.1, 1 µM) for 24 h followed by cell cycle analysis using flow cytometry. Representative cell cycle phases before and after treatment are shown as histograms. Percentage of cells in G0/G1, S or G2/M phases are displayed as bar graphs. Triplicates were used for each concentration. *Compared with the control group, inhibition was statistically significant (Student *t* test, p < 0.05). The effect of INC280 on CDK4, cyclin D3, p27, and γH2AX proteins was examined by western blot after cells were exposed to INC280 for 24 h at the indicated concentrations. **b** Sustained cell cycle arrest by INC280 in MKN45 cells. MKN45 cells were treated with INC280 for 24 h. The INC280 was removed by three PBS washes and the cells cultured in media without drug for another 24 or 48 h. At each time point, cells were harvested for cell cycle analysis using flow cytometry. Data is presented using both histograms and bar graphs as described above. **c** JHH5 and U87 cells were treated with INC280 (0, 0.1, 1 µM) for 24 h followed by cell cycle analysis using flow cytometry as described in **a**

To better understand the sensitivity of MET^*amp*^ cells in response to INC280 treatment, we analyzed for cell cycle arrest in treated MKN45 and MHCC97H cells using flow cytometry. We show that INC280 (0.1 and 1 µM) treatment for 24 h arrested these cells in G1 phase (MKN45 treated vs. control: 80.32% vs. 44.61%, p < 0.05; MHCC97H treated vs. control: 63.65% vs. 42.53%, p < 0.05, Fig. 2a). Because INC280 is a non-covalent inhibitor of MET, it is expected to fail to inhibit p-MET after a 24 h washout due to its dissociation from MET proteins or the formation of newly synthesized MET proteins that are not bound with INC280. However, we show that INC280 induced G1 arrest in MKN45 cells is sustained even after INC280 was removed for 48 h (Fig. 2b). Thus, the prolonged inhibitory activity did not likely result from the MET kinase inhibition, but by an irreversible injury caused by 24 h inhibition of MET signaling, i.e., DNA double strand breaks (DSB). In support of this conclusion, we found that INC280 up-regulated phosphorylation of histone 2A variant X (γH2AX), a hallmark of a DNA damage response (DDR), especially of DNA DSB, in a dose-dependent manner (Fig. 2a). In addition, INC280 treatment resulted in decreased levels of cyclin D3 and cyclin-dependent kinase 4 (CDK4), and increased expression of p27^*KIP1*^. In the cell cycle, the cyclin D/CDK 4/6 complexes are the activated checkpoint kinases controlling the transition from G1 into S phase [30]. The CDK inhibitory protein p27^*KIP1*^ binds to the cyclin D/CDK 4/6 complexes in quiescent cells (G_0_) to arrest them in the G_1_ phase [31]. In addition, in response to DNA damage, p27^*KIP1*^ can arrest cells in G_1_ and the other cell cycle phases by binding to the respective cyclin/CDK complexes [32, 33].

Compared with MKN45 and MHCC97H cells, the induction of G1 arrest by INC280 (1 µM) was less significant in JHH5 or U87 cells (JHH5 treated vs. control: 57.79% vs. 44.68%, p < 0.05; U87 treated vs. control: 40.58% vs. 31.62%, p > 0.05). Upregulation of γH2AX and p27^*KIP1*^ or downregulation of cyclin D3 or CDK4 was not observed (Fig. 2c). Our results suggest  that INC280 preferentially induces a DDR in cells propagating MET^amp^, leading to down regulation of cyclinD3 and CDK4 and up-regulation of p27^*KIP1*^, and ultimately arresting the cell cycle in the G1 phase.

### INC280-induced DSBs activate the p53BP1/ATM pathway for DNA repair in MET^amp^ cells

**Fig. 3:**
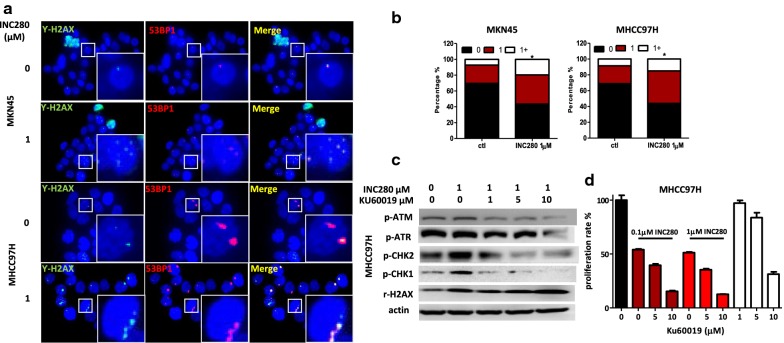
INC280 induces p53BP1 and γH2AX foci-formation in MKN45 and MHCC97H cells. MKN45 and MHCC97H cells were treated with vehicle or INC280 (1 µM) for 18 h and fixed for IF staining with γH2AX (green) or 53BP1 (red) antibodies and DAPI (blue) for nuclear staining. Triplicates were used for each concentration. Each co-localization of γH2AX and 53BP1 (yellow) is counted as one foci-formation at a DNA double-strand break. For each treatment at least 100 nuclei were counted based on fluorescence images (60×) to quantify foci formation. **a** Representative images showing the nuclei of cells with p53BP1 (red) and γH2AX (green) localization and foci formation (yellow) in MKN45 and MHCC97H cells. **b** The percentage of cells with 0, 1, and 1+ foci after vehicle or INC280 (1 µM) treatment for 18 h. *Compared with the control group, the increase of foci formation in the INC280-treated group was statistically significant (Chi square test, p < 0.05). **c** INC280 (1 µM) treatment for 24 h up-regulates the ATM/Chk1 pathway in MHCC97H cells and this up-regulation can be inhibited by the specific ATM inhibitor KU00019 as shown by western blot analysis. **d** Combination of INC280 and KU00019 treatment for 72 h results in a higher inhibitory efficacy in MHCC97H cells as measured by the CellTiter-Glo assay. Triplicates were used for each concentration. Data represents the mean ± SD for each treatment. Vertical bar represents the standard deviation

DSBs are the most harmful form of DNA damage and can be repaired by two different   mechanisms. Homologous recombination (HR) directs error-free DNA repair by using an unbroken sister chromatid as template to accurately restore the damaged DNA molecule. HR mainly occurs in S-phase, when the homologous sister chromatid is unwound for DNA replication. By contrast, nonhomologous end-joining (NHEJ) provides flexibility in repair by re-joining the broken ends together without critically proof-reading the DNA sequence, which also is a cause of chromosomal rearrangement [34]. An important regulator promoting NHEJ is p53-binding protein 1 (p53BP1) [34]. When DSBs occur, the ataxia telangiectasia mutated (ATM) signaling cascade is activated and drives p53BP1 to accumulate at the DSB sites; there it binds to γH2AX and forms foci [35]. The number of p53BP1/γH2AX foci is therefore indicative of DNA DSB repair [36]. While both MKN45 and MHCC97H parental cells showed a basal level of foci, INC280 treatment (1 µM) for 18 h significantly increased p53BP1/γH2AX foci number in  these cells  (Fig. 3a, b, Chi square test p < 0.05). Because the ATM pathway activates NHEJ to initiate DNA repair in G_1_ phase cells, we tested whether blocking ATM kinase activity may enhance the inhibitory efficacy of INC280 in cells with MET^amp^. We show that INC280 treatment alone induced p-ATM and downstream p-CHK1 and p-CHK2 in MHCC97H cells, which was reversed by ATM kinase inhibitor KU00019, resulting in a sustained γH2AX expression. At higher concentration (10 µM), KU00019 further up-regulated γH2AX expression, suggesting an enhanced DNA DSB effect (Fig. 3c). While the ATM inhibitor KU00019 alone at 1 or 5 µM displayed minor inhibition to cell proliferation, a combination with INC280 (0.1, 1 µM) increased inhibitory efficacy compared to INC280 alone (Fig. 3d). These results suggest that MET TKIs induce severe DSBs which undergo NHEJ repair in MET^amp^ tumors and that a combination strategy of using MET TKIs plus ATM inhibitors may enhance the therapeutic efficacy against tumor growth. With MKN45 cells, INC280 treatment induced up-regulation of p-ATM but not of downstream p-CHK2. Because INC280 at low concentration (0.1 µM) significantly inhibited cell proliferation, a combination of INC280 and KU00019 did not further enhance inhibition (Additional file 1: Figure S1).

### Endothelial cells are the secondary targets in HGF-autocrine tumors

To characterize the inhibitory efficacy in vivo, we subcutaneously transplanted MHCC97H and JHH5 cells into SCID mice and SCID*hgf*, a human HGF-transgenic SCID mouse strain with enhanced growth of human tumors in mice [5, 37]. These mice were treated with INC280 (daily oral gavage) or MetMab (one-time intraperitoneal injection). We show that both MHCC97H and JHH5 cells displayed similar tumor growth rate and responses to INC280 and MetMab in the two mouse models; thus, stroma cell-produced HGF does not play a significant role in MET^amp^ or HGF-autocrine tumor growth. MetMab potently inhibited JHH5 but not MHCC97H tumor growth, which is consistent with the in vitro results (Fig. 1) that MetMab only inhibit HGF-dependent MET activation.

**Fig. 4:**
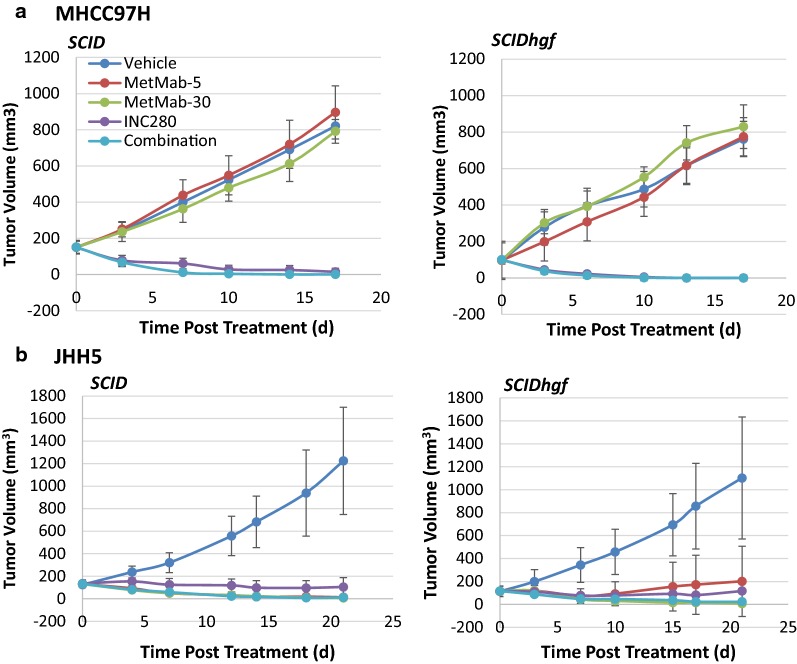
MHCC97H and JHH5 tumor growth inhibition by INC280 and MetMab in vivo. MHCC97H (**a**) or JHH5 cells (**b**) were implanted subcutaneously into SCID or SCID*hgf* mice to induce tumor growth followed by treatment with INC280 (30 mg/kg, daily oral gavage) for 3 weeks or MetMAb (5 or 30 mg/kg, i.p. one time dose). Combination refers to INC280 (30 mg/kg) plus MetAb at 5 mg/kg. Tumor growth was measured by a caliper twice a week

**Fig. 5:**
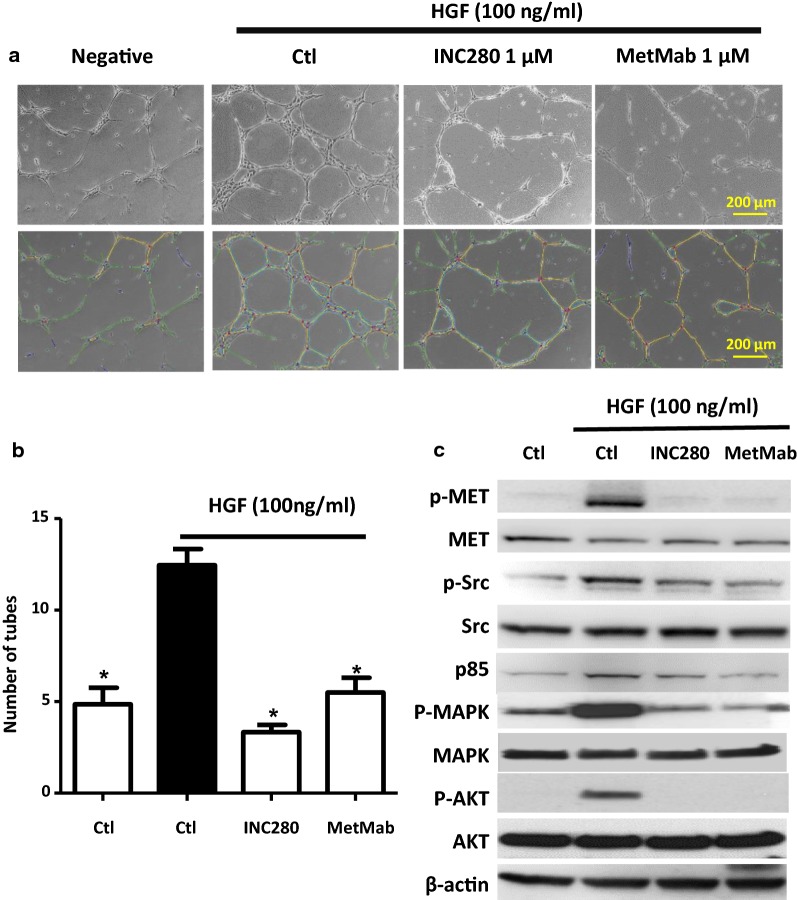
INC280 and MetMab inhibit HGF induced tube formation in HUVEC cells. **a** Representative images of HUVEC cell tube formation   treated with either INC280 (1 µM) or MetMab (1 µM). HUVEC cells were cultured in a 24-well plate coated with 50 μl Matrigel for the treatments as indicated. Triplicates were used for each treatment. After 16 h, cells were imaged by phase-contrast microscopy. Five images were taken randomly from each well. Tube formation before and after treatment are shown in upper and lower panel, respectively. **b** Quantification of tube-like structures as described above in **a**. The number of tubes is calculated as mean ± SD of 15 images that are measured by Image J. Fold change refers to the average number of tubes of each treatment group as compared to HGF stimulation alone group. Vertical bar refers to standard deviation. *Compared with the HGF stimulation control group, inhibition was found to be statistically significant (student *t* test, p < 0.05). **c** INC280 (1 µM) and MetMab (1 µM) inhibit phosphorylation of MET, MAPK/AKT and Src/p85 signaling pathway proteins in HUVEC cells as shown by western blot

MET^*amp*^ cells are more sensitive to INC280 than HGF-autocrine cells in vitro; however, MHCC97H tumors showed similar growth inhibition as JHH5, when treated  with INC280 (30 mg/kg, Fig. 4) in mice. These results raised two possibilities, either INC280 at high concentration (i.e., 30 mg/kg) can sufficiently block HGF-dependent MET activation in HGF-autocrine tumors at the same level as it does with MET^*amp*^ tumors, or, there are alternative targets other than the tumor cells that responded to INC280 in HGF-autocrine tumors. Because HGF is a potent angiogenic factor stimulating neovasculature formation by endothelial cells, we tested the effect of HGF-mediated 3-D HUVEC tube formation [28] for inhibition by either INC280 or MetMab (Fig. 5). We show that HGF at 100 ng/ml significantly stimulated HUVEC cell tube formation, which was significantly inhibited by either INC280 or MetMab (Fig. 5a, b). The Src tyrosine kinase signaling cascade is frequently activated during cancer progression, eliciting different cellular functions including—proliferation, adhesion and angiogenesis. Elevated Src kinase activity promotes endothelial cell tube formation which can be inhibited by Src inhibitors [38]. Here, we show that HGF stimulation upregulated Src/p85 signaling accompanied by MAPK and AKT activation in HUVEC cells (Fig. 5c). These HGF stimulatory effects were blocked by INC280 or MetMab. To verify the endothelial cells are secondary targets of MET inhibitors in HGF-autocrine tumors, we analyzed CD31 positive area using IHC staining with JHH5 tumors collected at day 3 of treatment (n = 3). We did not observe a statistical difference between the control and the treatment groups (data not shown), likely due to the time point being too early for changes in endothelial cells. Unfortunately, we were unable to analyze tumors collected at end point (day 21 of treatment) because at time of necropsy the tumors in treatment groups were too small to harvest. While we suggest that in HGF-autocrine tumors, both MET TKIs and neutralizing antibodies target not only tumor growth, but also HGF-mediated neovascularization via the Src signaling pathway in endothelial cells, further analysis of HGF-autocrine tumors from late stage treatment is necessary to validate the proposed “double hit” mechanism of inhibition.

## Discussion

We previously characterized two types of MET signal amplification in association with the MET pathway activities. Gain of an extra intact chromosome 7, which includes MET (MET^7gain^), is not biologically related to MET pathway  activity. In contrast, cells with MET^*amp*^, which have amplified copies of MET gene, are sensitive to MET inhibition [4, 15]. We also  showed that the total MET (tMET) expression level is not correlated with MET phosphorylation, and, therefore, may not be a sufficient marker for MET pathway activity [4, 15]. Here we show that INC280 but not MetMab significantly inhibits proliferation of MET^*amp*^ cells MKN45 and MHCC97H in vitro (Fig. 1) and tumor growth in vivo (Fig. 4). We previously reported that MHCC97H cells contain 25–100 copies of the MET gene per cell, together with MET translocations from chr.7 to chr.1 and chr.9 [4], likely resulting from DSBs and NHEJ repair. Notably, MET mutation and chromosomal rearrangement is a leading cause of MET oncogenic activation and malignant transformation [39, 40]. Both MKN45 and MHCC97H cell lines responded to INC280 but not MetMab, suggesting that MET^amp^-mediated MET overexpression, receptor dimerization, and signaling activation is more dependent on the intracellular tyrosine kinase domain than on the HGF/MET binding domain. Although we did not test whether increasing MetMab to higher concentrations may saturate MET binding, preventing its dimerization and signaling,  resulting in inhibition of MET^*amp*^ cell proliferation and tumor growth, our results do  suggest the use of neutralizing antibodies to inhibit HGF-dependent tumor growth. Note that one-time injection of MetMab at 5 mg/kg inhibited JHH5 tumor growth at the same level as INC280 daily oral dosing for 3 weeks, suggesting that neutralizing antibodies are a better choice than MET-TKIs in inhibiting HGF-autocrine tumors (Fig. 4). This also echoes the clinical trial results that MetMab, in combination with EGFR inhibitor erlotinib, failed to improve efficacy in a phase III lung cancer trial. After a phase II trial showing prolonged PFS and OS in MET-positive NSCLC patients [41], the phase III trial mainly used levels of tMET as a biomarker for patient selection. Taken together, we propose that MET^mut^ and MET^amp^, as well as HGF expression levels deserve evaluation in future clinical trials with MET inhibitors [27].

**Fig. 6:**
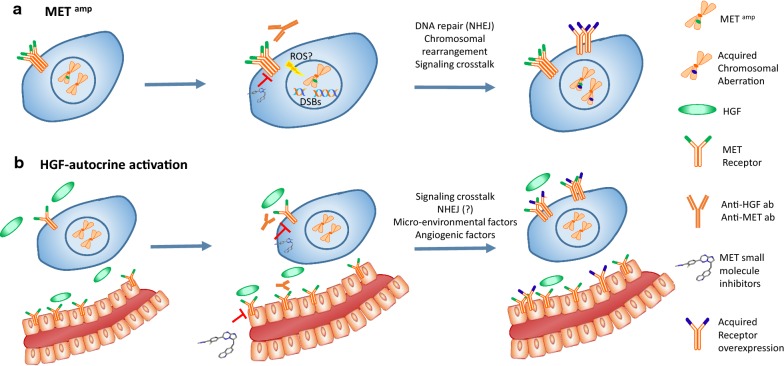
Proposed mechanisms of MET inhibitors in MET^amp^ and HGF-autocrine tumors. **a** MET^amp^ tumors are driven by receptor dimerization that is independent of HGF stimulation. They are sensitive to TKIs targeting MET intracellularly, but not to neutralizing antibodies interfering with extracellular ligand–receptor binding. In these tumors, constitutive inhibition of the MET signaling pathway may cause DSBs (i.e., via generation of reactive oxygen species, ROS) followed by DNA repair through the NHEJ process. Acquired resistance may occur through secondary chromosomal rearrangement via NHEJ. Combination of MET inhibitors with DNA repair inhibitors may enhance the therapeutic efficacy. **b** HGF-autocrine tumors are driven by endogenous HGF stimulation and are sensitive to both MET TKIs and neutralizing antibodies. Tumor-derived HGF further stimulates endothelial cells for neovasculature, which are the secondary targets in addition to the tumor cells. Acquired resistance may occur through MET signaling by-pass via other receptor tyrosine kinases, such as EGFR [48]; the micro-environmental response also plays an essential role. Combination with angiogenic inhibitors may enhance the therapeutic efficacy

Previous studies with PHA665752 demonstrated that MET TKI alone or in combination with ionizing radiation induced DNA DSBs and apoptosis in tumor cells [42]. As a key regulator in DNA repair following DSBs, p53BP1 binds to γH2AX at DSB ends and acts as a molecular scaffold that recruits additional DSB-response proteins. Mechanistically, p53BP1 amplifies ATM-dependent checkpoint signaling, especially at low levels of DNA damage, and promotes DNA repair through the NHEJ mechanism. NHEJ offers efficient rejoining of DSB ends but is an error-prone repair process as it may lead to chromosomal rearrangements [34, 43]. We show that INC280 treatment inhibited DNA synthesis, induced G1 phase arrest, upregulated γH2AX in MKN45 and MHCC97H (Figs. 1 and 2). These data indicate that MET amplification or translocation is sensitive to MET TKI-induced DNA double-strand break. While the increased number of γH2AX/p53BP1 foci indicates a repair mechanism employing NHEJ (Fig. 3), it also raises the speculation whether the frequency of rejoining DNA ends by NHEJ may become a cause of resistance. As evidenced in lung cancer studies, secondary MET^amp^ is found in EGFR T790M tumors that are resistant to the EGFR TKIs erlotinib or gefitinib [44], demonstrating that long-term treatment with TKI alone may allow NHEJ-mediated chromosomal rearrangement, leading to acquired resistance through alternative pathway activation (Fig. 6a). In our study, we show that INC280 treatment alone activates ATM signaling and that a combination of INC280 and KU00019 increased inhibitory efficacy more than INC280 alone (Fig. 3d). The combination failed to improve the inhibitory efficacy in MKN45 cells, suggesting a strong MET oncogenic addiction and that INC280 alone is sufficient in inducing G1 arrest followed by apoptosis, at least at an early stage of treatment. As such, it would be worthwhile to induce resistance to INC280 in MKN45 and further analyze the ATM activation and inhibition. Interestingly, as part of the DDR, activated ATM phosphorylates p27^*KIP1*^ at Ser140 in the G1 phase and stabilizes p27^*KIP1*^, thus enhancing G1 arrest [32]. This is consistent with our results that INC280 increased levels of p27^*KIP1*^ as seen in Fig. 2 and, perhaps, also explains the decreased levels of CDK4 and cyclin D3 to which the p27^*KIP*^ binds. Thus, stabilizing p27^*KIP1*^ or adding CDK4 inhibitors also may improve the efficacy of INC280 in MET^amp^ cells. Taken together, our results suggest that the combination of MET and ATM inhibitors may enhance the therapeutic efficacy and prevent resistance for treating tumors with MET chromosomal rearrangements, such as amplification, translocation and/or mutation (Fig. 6a).

During cancer progression, tumor cells often produce cytokines and growth factors to remodel the host tumor microenvironment to facilitate their growth. In particular, endothelial cells perform a major role in neovasculature formation, and are essential to satisfy the need for increased blood supply during rapid tumor growth. Because HGF is a pleiotropic cytokine that also promotes endothelial cell proliferation, migration, and capillary formation, tumor cell-derived HGF in autocrine tumors may stimulate vascular formation via paracrine activation. Previous studies have shown that HGF can upregulate VEGF mRNA and protein expression in endothelial cells, and that a combination of the two factors enhanced the anti-angiogenic efficacy, either additively or synergistically [45, 46]. In liver cancer, overexpression of HGF promotes carcinogenesis through an HGF-autocrine mechanism, resulting in high levels of neovascularization mediated by dynamic interaction between the endothelial and tumor cells [47]. When rapid tumor growth depletes local blood supply, long-term adaptation to hypoxia occurs through angiogenesis promoted by the HGF/MET axis [48]. In addition, HGF also protects cells from hypoxia-mediated endothelial damage and apoptosis by upregulating Bcl-2 and Bcl-xL [49]. Here we show that HGF induces HUVEC tube formation which is inhibited by either INC280 or MetMab through the MAPK/AKT and Src signaling pathways. As such, in HGF-autocrine tumors, MET inhibitors target both tumor cells and endothelial cells. Given that vasculature formation is a complex process involving variable growth factors and cytokines, combination of angiogenesis inhibitors with MET inhibitors may enhance the therapeutic efficacy for treating HGF-dependent tumors (Fig. 6b).

## Conclusion

After several clinical trial failures, application of MET inhibitors needs to be guided by predictive biomarkers to restore confidence in their use [27]. Understanding the mechanism of MET inhibitors is, therefore, the key to the development of patient stratification strategies and tailored combination therapeutics. Here, we characterized tumors harboring MET^*amp*^ or HGF-autocrine activations and sub-classified their therapeutic responses to MET TKIs or neutralizing antibodies. We show that MET^*amp*^ indicates a predisposition to a DNA DSB response, by which therapeutic efficacy may be enhanced by combination with inhibitors of ATM or other DNA repair proteins. In fact, tumor heterogeneity further blunts the drug response. In the case of multiple receptor tyrosine kinase amplifications within the same tumor, loss of function of tumor suppressors may be the original cause and a target of therapeutic combinations via the synthetic lethal approach [7].  By contrast, HGF-autocrine tumors are less sensitive to DNA damage but are more angiogenic due to HGF-mediated neovascularization. As such, a combination with angiogenesis inhibitors should be considered to improve the therapeutic efficacy of MET inhibitors.

In addition to proliferation and angiogenesis, MET activation is known to promote cancer invasion and metastasis [3, 40]. Thus, it is worthwhile to determine whether INC280 prevents MHCC97H metastasis at similar level as it inhibits cell proliferation in vivo. Given the recent breakthrough in cancer immunotherapy, understanding the role of MET inhibition in tumor immunology is essential toward future improvement of MET-targeting therapeutics. As cabozantinib, a multi-target kinase inhibitor targeting MET and VEGFR2, has shown positive clinical trial results in improving survival in advanced HCC [24], combining MET inhibitors with immune checkpoint inhibitors to improve therapeutic efficacy are certainly the future directions.

## Additional file

**Additional file 1: Figure S1.** INC280 alone is sufficient in inhibiting MKN45 proliferation. **(A)** INC280 (0.1, 1 µM) treatment for 72 hrs potently inhibited MKN45 proliferation as measured by the CellTiter-Glo assay. Further combination with ATM inhibitor Ku60019 did not improve efficacy. Data represents for Mean ± SD. Vertical bar represents the standard deviation. **(B)** INC280 mediated ATM signaling pathway activity in MKN45 cells.

## Abbreviations

ATM: ataxia telangiectasia mutated
CDK4: cyclin-dependent kinase 4
DDR: DNA damage response
DSB: double stand break
GBM: glioblastoma
HCC: hepatocellular carcinoma
HGF: hepatocyte growth factor
HR: homologous recombination
HUVECs: human umbilical vein endothelial cells
MET^*amp*^: MET amplification
MetMab: Met monoclonal antibody
NHEJ: nonhomologous end-joining
NSCLC: non-small cell lung cancer
OS: overall survival
PRCC: papillary renal cell carcinoma
PFS: progression free survival
p53BP1: p53-binding protein 1
γH2AX: histone 2A variant X
TKI: tyrosine kinase inhibitor
