# Supplementary material for: Differential responses of MET activations to MET kinase inhibitor and neutralizing antibody
Source: J Transl Med. 2018 Sep 12;16:253. doi: 10.1186/s12967-018-1628-y (PMC6134500; doi:10.1186/s12967-018-1628-y)
Supplement: Supplementary file 1 — Additional file 1: Figure S1. INC280 alone is sufficient in inhibiting MKN45 proliferation. (A) INC280 (0.1, 1 µM) treatment for 72 hrs potently inhibited MKN45 proliferation as measured by the CellTiter-Glo assay. Further combination with ATM inhibitor Ku60019 did not improve efficacy. Data represents for Mean ± SD. Vertical bar represents the standard deviation. (B) INC280 mediated ATM signaling pathway activity in MKN45 cells. [file 12967_2018_1628_MOESM1_ESM.docx]

**Differential Responses of MET Activations to MET Kinase Inhibitor and Neutralizing Antibody**

Jianqun Kou^1,2^, Phillip R. Musich^1^, Ben Staal^3^, Liang Kang^3^, Yuan Qin^1,2^, Zhi Q. Yao^2,4^, Boheng Zhang^5^, Angela Tam^6^, Alan Huang^6^, Huai-Xiang Hao^6^, George F. Vande Woude^3^ and Qian Xie^1,2*^

^1^ Department of Biomedical Sciences, Quillen College of Medicine, East Tennessee State University, Johnson City, TN 37614

^2^ Center of Excellence for Inflammation, Infectious Disease and Immunity, Quillen College of Medicine, East Tennessee State University, Johnson City, TN 37614

^3^ Center of Cell and Cancer Biology, Van Andel Research Institute, Grand Rapids, MI 49503

^4^ Department of Internal Medicine, Quillen College of Medicine, East Tennessee State University, Johnson City, TN 37614

^5^ Liver Cancer Institute, Fudan University Zhongshan Hospital, Shanghai, 200032, China

^6^ Novartis Institutes for BioMedical Research, Cambridge, MA 02139

**Corresponding Author:**

Qian Xie, Department of Biomedical Sciences, Quillen College of Medicine, East Tennessee State University, Johnson City, TN 37614

Email: [xieq01@etsu.edu](mailto:xieq01@etsu.edu)

Tel: 423-439-5332

**Conflict of Interest:** The authors declare no conflict of interest.

**Data Supplement has one Supplementary Figure.**

**A**

**MKN45**

**B**

**INC280 µM**

**KU60019 µM**

**0 1 1 1 1**

**0 0 1 5 10**


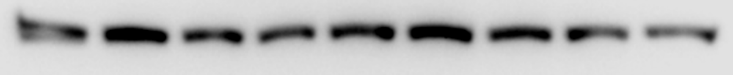

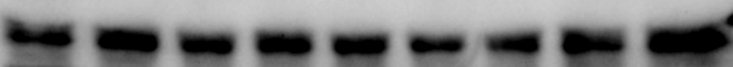

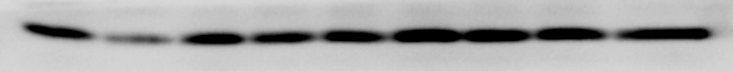


**p-ATM**

**p-CHK1**

**p-ATR**

**p-CHK2**

**ϒ-H2AX**

**MKN45**


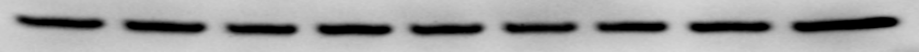


**β-actin**


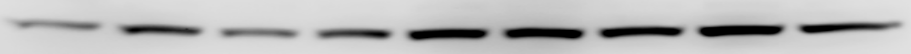

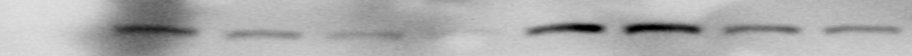


**Supplementary Figure 1. INC280 alone is sufficient in inhibiting MKN45 proliferation.**

**(A)** INC280 (0.1, 1 µM) treatment for 72 hrs potently inhibited MKN45 proliferation as measured by the CellTiter-Glo assay. Further combination with ATM inhibitor Ku60019 did not improve efficacy. Data represents for Mean±SD. Vertical bar represents the standard deviation. **(B)** INC280 mediated ATM signaling pathway activity in MKN45 cells.
